# Estimation of Soil Organic Carbon Storage in Palustrine Wetlands, China

**DOI:** 10.3390/ijerph17134646

**Published:** 2020-06-28

**Authors:** Lu Han, Zhongmei Wan, Yuedong Guo, Changchun Song, Shaofei Jin, Yunjiang Zuo

**Affiliations:** 1 College of Earth Sciences, Jilin University, Changchun 130061, China; hanl17@mails.jlu.edu.cn; 2 Key Laboratory of Wetland Ecology and Environment, Northeast Institute of Geography and Agroecology, Chinese Academy of Sciences, Changchun 130102, China; songcc@iga.ac.cn (C.S.); zuoyunjiang@iga.ac.cn (Y.Z.); 3 Department of Geography, Ocean College, Minjiang University, Fuzhou 350108, China; jinsf@tea.ac.cn; 4 College of Resources and Environment, University of Chinese Academy of Sciences, Beijing 100049, China

**Keywords:** palustrine wetlands, soil organic carbon storage, spatial distribution, environmental factors

## Abstract

Wetlands regulate the balance of global organic carbon. Small changes in the carbon stocks of wetland ecosystem play a crucial role in the regional soil carbon cycle. However, an accurate estimation of carbon stocks is still be debated for China’s wetlands ecosystem due to the limitation of data collection and methodology. Here, we investigate the soil organic carbon (SOC) storage in a 1-m depth in China’s palustrine wetlands. A total of 1383 sample data were collected from palustrine wetlands in China. The data sources are divided into three parts, respectively, data collection from published literature, data from books, and actual measurement data of sample points. The results demonstrate that there is considerable SOC storage in China’s palustrine wetlands (9.945 Pg C), primarily abundant in the northeast, northwest arid and semi-arid as well as Qinghai-Tibet Plateau regions. The SOC density in per unit area soil was higher in the wetland area of northeast, southwest and Qinghai-Tibet plateau. Within China terrestrial scale, the temperature and precipitation differences caused by latitude were the main environmental factors affecting the organic carbon content. Furthermore, except for the southeast and south wetland region, SOC content decreased with depth.

## 1. Introduction

Wetlands are important ecosystems located at the water-land interface, and have a significant effect on regulating the source-sink mechanism of soil organic carbon (SOC) [1]. With respect to optimal water-incubated conditions [2], wetlands, which only account for 5–8% of terrestrial land [3,4], contain 20–30% of soil organic carbon pool [5]. Palustrine wetlands refer to marshes, swamps, bogs, and fens, and are the major wetlands types investigated in this study [6]. In China, palustrine wetlands were primarily distributed in the northeast, southwest and south temperature-incubated regions, as well as the northwest and coastal water-incubated regions [7,8]. Numerous studies have demonstrated that there are various differences in SOC storage enriched in different geographic regions, which is strongly influenced by vegetation type, landscapes and hydrological conditions [9,10]. For instance, experiments in Wang et al. [11] indicated that SOC storage in tropical mangrove wetlands varies by 1–8 times along the tidal flooding gradient. Additionally, Li et al. [12] determined that long-term centralized management distinctly reduces SOC storage. Further, Liu et al. [13] concluded that soil carbon dioxide (CO_2_) shows seasonal changes with routine. Although, many studies have been conducted to identify the physical, chemical and biological factors on SOC biogeochemical circulation, only a few parameters in these analyses could account for the correlation between SOC and soil properties on a national-scale [14,15,16]. Thus, a thorough understanding on SOC biogeochemical circulation is needed.

At present, there are still many uncertainties in SOC estimations, which limits the understanding of the important role of wetlands in the global carbon circulation and climate change mitigation. Firstly, the total wetland area has been reduced by 68% due to climate change and anthropogenic activities [10,17], resulting in uncertainty in SOC estimations on regional and global scales [18]. Secondly, using the average peat layer thickness of 2.3 m, the estimated global wetland SOC storage is 445 Pg [19], which is nearly 2-times higher than earlier estimate (225 Pg) with the average peat layer thickness of 1.0 m [18]. Finally, scholars believe that the variability of wetland carbon stocks in different climate zones is evident [9,10], and thus the practicality of estimation methods needs further verification. Therefore, conducting a research on SOC storage at a national-scale will advance our understanding of spatial variation in SOC.

Despite considerable efforts directed at understanding SOC distribution and storage on global [20,21], country [22,23], and regional [24] scales, numerous studies primarily focused on agro-ecosystems [25,26], forest-ecosystems [27,28], grassland-ecosystems [29,30], while an accurate understanding of the distribution and storage of SOC in national-scale marshlands in still lacking. The spatial distribution of carbon storage in palustrine wetlands is affected by different factors from forests and farmland. The original theory suggests that the development process of wetlands is mainly affected by small-scale geomorphologic and hydrological processes [31] and has non-zonal characteristics. However, wetland productivity is also affected by solar energy on a large scale. Therefore, the distribution characteristics of wetland carbon reserves on a national scale, and whether there is a significant latitude effect, needs to be further discussed and verified. To address these issues, the central objectives of this study are threefold: (1) to investigate the SOC storage in China’s palustrine wetlands; (2) to identify the spatial and vertical distribution with profile depth in different geographic regions; and (3) to analyze the dominant environmental controls on SOC content at regional and national scales.

## 2. Database and Methods

### 2.1. Data Collection

**Figure 1:**
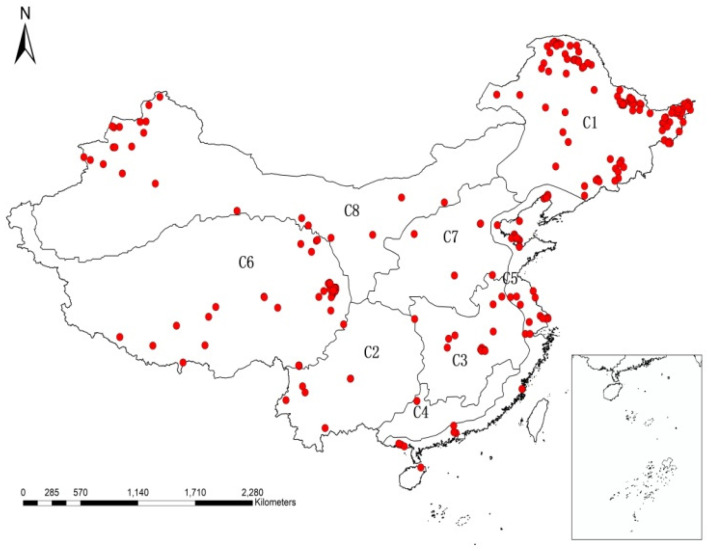
Selected sampling points distribution diagram.

In order to ensure the validity and accuracy of the data, the data sources of this paper are divided into three parts: literature data collection, field survey and measurement data, and preliminary data sorting. A systematic literature search was conducted with Web of Science, Elsevier Science Direct, Google Scholar and China National Knowledge Infrastructure using the following keyword combinations ‘marsh wetland’, ‘swamp wetland’, bogs’, ‘fens’, and ‘soil organic carbon OR soil organic matter’ in the title, abstract and keywords. The research on carbon storage in China’s palustrine wetland began in the late 1990’s, limiting this study to a more recent timespan which was represented in the literature. The oldest relevant paper was published in 2000, while the most recent research was published in 2019. A total of 1267 studies were entered into databases and analyzed for relevance. Articles which were deemed irrelevant or repetitive were exclude during a preliminary screening procedure, while the remaining publications were evaluated to extract data on SOC or SOM and the influencing environmental factors, including longitude, latitude, altitude, mean annual precipitation (AMP), mean annual temperature (AMT), moisture content (MC) and acid-base property (pH). To improve the spatial distribution of data points extracted from the literature, historical data and additional measured datapoints are used in supplement to this paper. Historical data was obtained from the book “China Marsh Annals”, from which data including relevant information of longitude, latitude, soil organic carbon content, soil bulk density, water content and pH of each point was extracted, totaling 67 sample points. However, the distribution of data from literature and historical resources is considerably lower in wetlands in the Great Hing’an Mountains, in the northeast of China. The Great Hing’an Mountains area is located along the southern margin of the Eurasian tundra, and is sensitive to climate change. Therefore, study on the carbon storage of palustrine wetlands in this area is of great importance for the estimation of SOC in China’s palustrine wetlands. Given this, 49 effective samples were collected in the Great Hing’an Mountains to improve the spatial distribution of the data. In addition, in order to verify the accuracy of the mathematical model use in this paper, three 1-m soil cores were collected in each wet area for error analysis. The distribution of sample points was shown in Figure 1.

Based on the distribution of wetlands in China, and considering the natural variation in these characteristics across different regions (e.g., hydrological and geomorphic features, wetland functions, etc.), China’s wetland resources are divided into eight wetland regions, the northeast wetland region (C1), the southwest wetland region (C2), wetlands in the middle and lower reaches of the Yangtze River region (C3), the southeast and south wetland regions (C4), coastal wetland region (C5), wetlands in the Qinghai-Tibet plateau region (C6), wetlands in the middle and lower reaches of the Yellow River regions (C7) and wetlands in the northwest arid and the semi-arid regions (C8). Further information on geography of these regions is showed in Table 1.

### 2.2. Methodology

As an indicator of soil physical structure, bulk density (BD), which is related to the water holding capacity of soil and SOC biogeochemical circulation, was used in the construction of soil mathematical model [32,33]. In theory, BD can be evaluated through dividing the dried weight of soil by the total volume, but to undertake measurements of BD on a large scale is labor-intensive, time-consuming, and cost-expensive [34,35]. As a result, BD measurements are often missing from soil databases. To overcome this issue, Han et al. [36] used a stepwise multiple regression procedure and established two simulation equations (Equations (1) and (2)) for estimating BD calculating from SOM or SOC content using 1560 sampling points. However, China has a vast territory and diverse soil types, so the applicability of these transfer functions for BD to different soil ecosystems is also worth exploring. In 2016, Han et al. [37] evaluated the prediction accuracy and applicability of the afore mentioned simulation equations in different soil classes and proposed a modified relationship equation between BD and SOM (Equation (3)), which holds true to gleysols. In palustrine wetlands, soil is characterized as bog soil incubated via the processes of submergence, septic, and peatification. Thus, missing BD values were calculated using Equation (3). Simulation results shown that predicted values presented an acceptable error compared to measured values.

(1) $$\mathrm{BD}=\frac{0.167\times 0.526}{1.526\times \mathrm{SOM}+0.167\times \left(1-\mathrm{SOM}\right)}$$

(2) $$\ln\mathrm{BD}=0.5379-0.0653\times {\mathrm{SOM}}^{0.5}$$

(3) lnBD=0.215−0.0025×SOM+0.0017×H

where, BD (g/cm^3^), SOM (g/kg) and H (m), represent soil bulk density, soil organic matter content and soil depth. In this study, the downcore was divided into five layers: 0–20, 20–40, 40–60, 60–80 and 80–100 cm).

Soil organic carbon density (SOCD_i_) per unit area is estimated for each layer of i [38] and is derived from BD (Equation (4)) [39,40].

(4) $${\mathrm{SOCD}}_{i}=C_{i}\times {\mathrm{BD}}_{i}\times H_{i}$$

where C_i_ and BD_i_ represent SOC content and BD within a layer of i, and H_i_ represents the depth of the soil in that layer.

In this study, only SOC or SOM content in the soil surface layer was available, while the vertical distribution of SOC content is also essential information for estimating SOC storage. Numerous studies demonstrated that SOC content decreased rapidly with depth as a function of soil types, land-use, and vegetation communities [38]. In recent years, many measured data fitting results indicated that the changes in SOC content associated with profile depth can be quantitatively described in a form of equation, such as negative exponential function [26,41] (Equation (5)), power function [42] and logarithmic function [43]. By comparing the error analysis between the predicted value and the measured value, we selected negative exponential function to estimate SOC content in the lower layer. The parameters of the aforementioned equations can be estimated simple soil properties to overcome the shortage of sample data. Subsequently, SOC content per unit volume of 1 m depth was obtained with integral method, and SOC density was multiplied by the area (Equation (6)).

(5) $$S-\mathrm{SOC}=C_{a}\exp\left(-k\times H\right)+C_{b}$$

(6) $$T-\mathrm{SOC}\;=\overset{5}{\underset{1}{\sum }}\left[\left(S-\mathrm{SOC}\right)\right]\times S$$

where C_a_, C_b_, and K were constants calculated from exponential function to calculate a region’s SOC storage (T-SOC, kg) per unit area (S, m^2^). The specific information about each area is shown in Table 1.

Most of the SOC data collected was from surface layers with a few datapoints from deeper layers (1 m). To ensure the reliability of the mathematical model, we selected some data to verify the mathematical model. The verification data were divided into two parts: one was the measured SOC content at 1 m depth collected from published literature; the second part was from the survey undertaken at the sample site. We sampled each wetland regions, collecting 1 m core of undisturbed soil which was analyzed in the laboratory for measurement.

**Figure 2:**
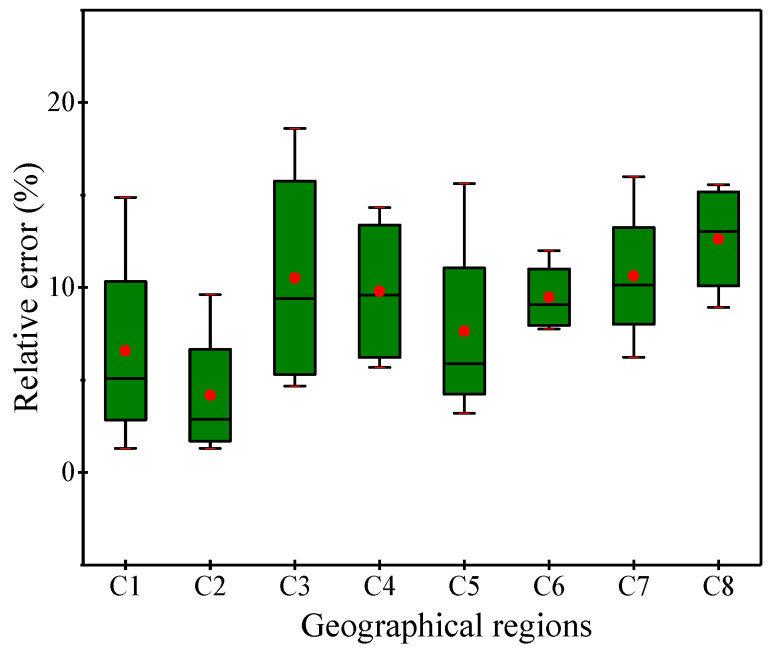
Relative error analysis between SOC measured value and estimated value.

Several measured values were selected to verify the applicability of equation, and demonstrated a maximum error of 18.61%, a minimum of 1.31%, and a mean error of 8.93% between measured and estimated SOC values (Figure 2).

### 2.3. Statistical Analysis

Experimental data was processed with Excel software Ver. 2007 (Microsoft, Redmond, WA, USA) to calculate BD. Redundancy analysis (RDA) was completed with Canoco software Ver. 4.5 (Biometris-Plant Research International, Wageningen, The Netherlands) to identify the explanatory capacity of environmental variables to SOC content. Using the statistical software SPSS Ver. 20.0 (SPSS, Chicago, IL, USA), the direct and indirect influence of environmental variables on SOC distribution were assessed with path analysis (PA). Structural equation models (SEMs) was conducted with Amos Ver. 20.0. All figures were generated using Origin Ver. 9.0 (Microcal, Malvern, UK).

## 3. Results and Analysis

### 3.1. Simulated SOC Storage

**Figure 3:**
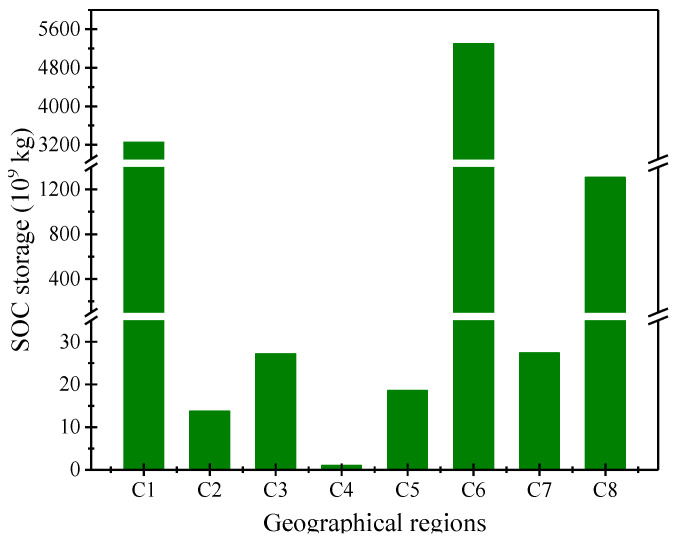
SOC storage in eight geographic regions.

Results showed there is a considerable SOC storage in China’s palustrine wetlands, with a total of 9.945 Pg (Figure 3). However, significant difference was observed across the eight geographic regions. The carbon storage of palustrine wetlands in China is mainly distributed in Qinghai-Tibet plateau, the northeast and northwest of China. SOC reserves in these regions accounted for 53.28%, 32.69%, and 13.14% of the total reserves in all marsh and wetland, respectively, accounting for 99.11% in total. In C1, C6 and C8 geographic regions, SOC storage was high at 3.251, 5.299, and 1.307 Pg C, respectively; while C2, C3, C5, and C7 geographic regions had 0.0137, 0.0272, 0.0185, and 0.0274 Pg C, respectively; The lowest SOC at only 0.0009 Pg C was measured in the C4 geographic region. Specially, a total of 3.456 Pg C was estimated with a depth profile of 0–0.3 m.

**Figure 4:**
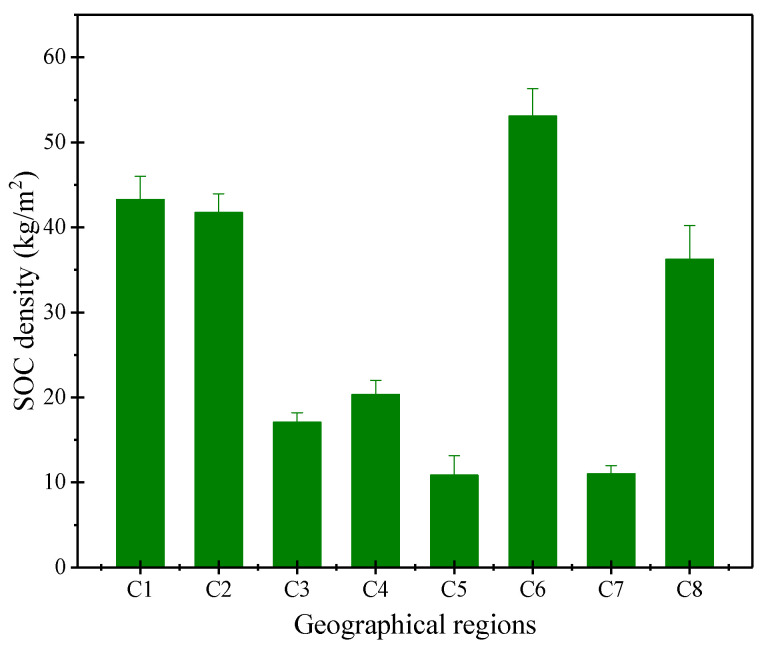
SOC density in the eight geographic regions.

SOC density showed clear variation between geographic regions (Figure 4). In the C1, C2, C6, and C8 geographic regions, SOC density was higher with values of 43.29, 41.71, 53.06, and 36.23 kg/m^2^, respectively, followed by the C4 (20.31 kg/m^2^), C3 (17.04 kg/m^2^), C7 (10.98 kg/m^2^), and C5 (10.84 kg/m^2^) geographic regions. The average SOC density was 27.17 ± 16.54 kg/m^2^.

**Figure 5:**
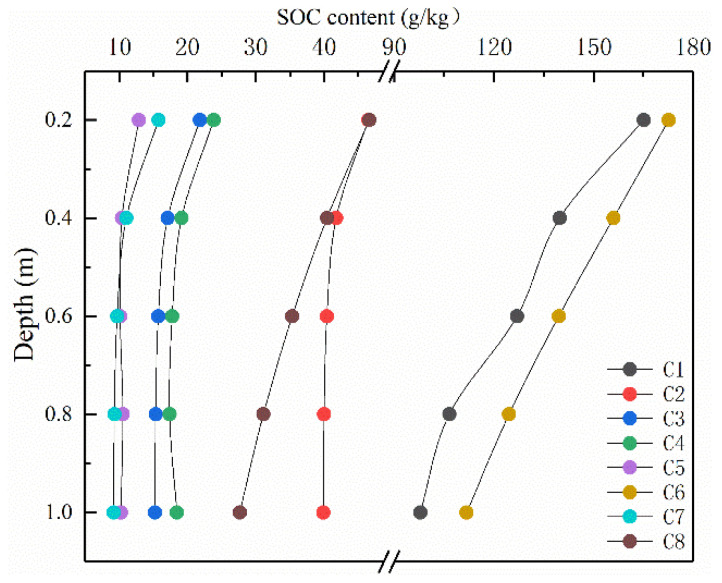
Changes in SOC content with soil depth for each geographic region.

With respect to SOC storage and content, the C6 and C1 geographic regions showed higher values, particularly C6 (Figure 5). SOC storage and content showed irregular trends due to the spatial distribution of sample points. With exception of the C4 geographic region which showed an SOC content increase the sub-layer of 0.8–1.0 m, a continuous decrease was observed in all other geographic regions. This phenomenon can be interpreted as soluble organic carbon accumulates in the bottom layer of the soil because water migrates to deep layer following precipitation. SOC content in each sub-layer was higher in the C6 geographic region, followed by C1, C2, C8, C4, C3, and the lowest SOC content in the C5 and C7 geographic regions. Although SOC content in the surface layer was relatively higher in the soils from the C6, C2, C1, and C8 geographic regions, SOC showed a rapid decrease with soil depth.

### 3.2. Environmental Controls on SOC Content

RDA showed that the first two axis components explained 76.10% of the variance in the correlation between SOC content and environmental controls (Table 2). More than 57.90% of the change in SOC content was explained by the combination of longitude, latitude, altitude, MAP, MAT, MC, and pH, indicating that environmental controls identified in the literature have a significant influence on SOC biogeochemical circulation. Of these, MAT was identified as the factor which explained the most amount of variance (23.74%), followed by longitude (18.2%), latitude (7.12%), altitude (5.02%), MAP (3.75%), and finally pH (0.75%).

Best-fit stepwise regression results indicated that when combined, these selected environmental controls can account for 99.9% variation in SOC content (Table 3). Latitude had a positive effect on SOC content with a direct path coefficient of 0.856. Meanwhile, MAP and MAT variation as a function of latitude had a negative influence on SOC content with an indirect path coefficient of −0.594 and −0.659, respectively. The direct path coefficients of MAP and MAT was 0.823, and was the second most significant environmental factors which influenced SOC content. PA results showed that the dominating environmental factors related to SOC content was latitude, followed by MAP, MAT, longitude, altitude, MC, and pH. In summary, the difference in MAP and MAT caused by latitude was the main reason for the spatial differentiation in SOC content. Notably, in the C1 geographic region, MAT presents a significant effect on SOC content, while no similar result was observed in the C6 geographic region. However, pH and MAP generally (*p* < 0.05) and significantly (*p* < 0.01) correlated with SOC content in both C1 and C6 geographic regions.

**Figure 6:**
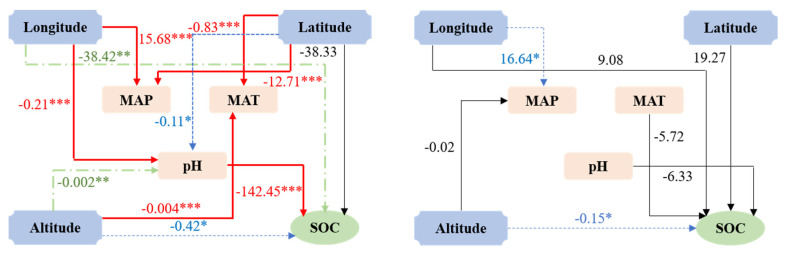
SEMs, representing hypothesized causal relationships between SOC content and physio-chemical parameters. Note: “*” effect is significant at *p* < 0.05 level; “**” effect is significant at *p* < 0.01 level; “***” effect is significant at *p* < 0.001 level.

Due to the significant differences in climate characteristics, topography and geomorphology in the different geographical areas, the migration and transformation of SOC are affected by different environmental factors. In this study, the typical wetland regions of northeast China and Qinghai-Tibet plateau were selected for analysis (Figure 6). The results of the structural equation model (SEMs) show that in the wetland regions of northeast China, the pH difference caused by longitude and altitude is the main environmental factor affecting SOC content. The SOC content in the wetland regions of Qinghai-Tibet plateau is mainly controlled by altitude.

## 4. Discussion

### 4.1. Spatial Distribution and Characteristics of SOC on a National-Scale

With respect to wetland formation, palustrine wetlands is the manifestation of bidirectional ecological evolution processes between the land and surface water, which is formed under the comprehensive action of various natural factors, such as regional climate, hydrology, geological landform, and vegetation [44]. Climate is an important factor affecting the global biochemical cycle and plays an important role in the accumulation of organic carbon in the wetland ecosystem. Precipitation and air temperature are very important environmental factors affecting the local climate. Climate changes caused by water and temperature will have a significant impact on the input and decomposition of organic carbon in wetland soil. Firstly, climate conditions restrict the types of vegetation and affects the productivity of the ecosystem, thus affecting the input of SOC. Secondly, soil microorganisms are the main driving force for the decomposition and transformation of SOC. Climate affects the activity of microorganisms by affecting the hydrothermal conditions of soil, thus affecting the decomposition and transformation of SOC by microorganisms. In this study, SOC content was higher in the C1, C2, C6, and C8 geographical regions. This result can be explained by the complex influence associated with wetland development, climatic characteristic, and vegetation abundance. However, SOC storage showed a disparate spatial trend compared to SOC content. SOC storage was primarily distributed in the C1 and C6 geographical regions, which maybe contributed to the high density of SOC and large spatial distribution. Generally, SOC content is closely related to its parent materials on a national-scale. Except for the Qinghai-Tibet Plateau, in the south of the Qinling-Huaihe region of China, it belongs to the subtropical to tropical climate. Due to the influence of the humid monsoon, soil types such as yellow-brown soil, yellow soil, krasnozem, and latosol appear from north to south. In these areas, soil leaching is intense and the soil is more acidic (pH = 5.0–5.5) [45], which inhibits the influence of microorganisms and hence the accumulation of SOC. In the north of the Qinling-Huaihe region, from south to north there is, a transition from a brown soil to a black soil region. Typical black soil is derived from river-lake sediments and enriched with carbon [46], resulting in higher SOC content in these palustrine wetlands. In general, SOC distribution in zones with latitude across China. However, due to the complexity and diversity of China’s climate and terrain, in some small areas, there are still non-zonal features that are affected by the integrated environment.

Climatic factors, especially hydrothermal conditions, are an important influence on the formation and development of palustrine wetlands and indirectly regulate SOC source-sink processes through affecting vegetation community assemblage and growth, as well as plant-residue decomposition quality and intensity [47]. The considerable SOC content in the C1, C2, and C6 geographical regions may be attributed to the control of temperature. High temperature accelerates vegetation to trap more carbon dioxide (CO_2_) from the atmosphere [48], enhancing plant photosynthesis; this can explain why SOC content in the C2 geographical region was higher, while higher SOC content in the C1 and C6 geographical regions was due to low temperatures which inhibit microorganisms and enzyme activity, reducing the decomposition rate of plant residues and facilitating SOC accumulation [49]. China’s tropical and subtropical regions have a hot climate with high temperatures, and large quantities of vegetation in the marshlands; however, the temperature is too high, which accelerates decomposition of vegetation residues, and inhibits SOC accumulation. Additionally, from cold temperate zones to the tropical zones, vegetation respiration increases, as seen in the results from the C3, C4, and C7 geographical regions which showed lower SOC content than that in the C1 geographical region. Explicitly, SOC storage in marsh is dominated by the amount of vegetation litter and decomposition [50].

### 4.2. Environmental Factors Controlling SOC Content

Soil physical properties, such as MC and BD [51,52], and biological properties including pH and microbial communities [15,53], can control SOC spatio-temporal distribution through regulating SOC accumulation-decomposition, precipitation-dissolution, adsorption-desorption, and oxidation-reduction process [54,55]. In the C1 and C6 geographic regions, SOC content per unit volume was higher than that in the C2, C3, C4, and C7 geographic regions. This phenomenon could be attributed to the difference in the temperature caused by latitude and altitude, confirming the conclusion that reduced temperature can restrict SOC mineralization and accelerate SOC accumulation [56,57]. Two possible reasons for this result include that lower temperatures inhibited soil microbial communities, resulting in a decrease in SOC mineralization rate [58], or that, compared to hotter geographic regions, plants, and animals respiration rate is significantly lower than that in colder geographic regions, leading to a decrease in CO_2_ emissions [13]. Additionally, decomposition rate of plant residues by soil microorganisms was relatively lower in the C1 and C6 geographic regions, resulting in an increase in SOC sources.

Generally, MC plays an important role in the SOC source-sink process through regulating the oxidation-reduction potential (ORP), microbial biomass and vegetation types [48,59,60]. Heikkinen et al. [61] demonstrated that anaerobic environments inhibited SOC mineralization and biological respiration, which accelerated the SOC accumulation. This is seen in the results presented in this paper which showed that SOC content per unit volume in the C2 geographic region was higher than that in the C3 and C7 geographic regions. On the other hand, SOC content per unit volume in the C8 geographic region was markedly higher compared to C3, C4, and C7 geographic regions, which may be attributed to the fact that the climate of this region is characterized by frequent droughts and less biomass, so the disturbance of original SOC is small. Experiments in Sun et al. [62] and Meng et al. [63] demonstrated that the binning points on the surface of fine-aggregates are occupied by metal ions under highly-saline condition, thus releasing abundant dissolve organic carbon (DOC), which provides more available carbon sources for the mineralization and decomposition by soil microorganisms. Similarly, the surface soil of coastal wetlands is significantly eroded by flowing waters, which in turn are inundated with seawater, thus causing the loss of a large amount of dissolved organic carbon (DOC) by seawater erosion. This corresponds results presented here, which showed that SOC content in coastal wetlands (C5 geographic region) was lower than that in other geographic regions.

## 5. Conclusions

There was a considerable SOC storage in China’s palustrine wetlands with a value of 9.945 Pg C, primarily abundant in the northeast and northwest arid and semi-arid regions as well as Qinghai-Tibet Plateau regions, account for 99.11% of total SOC storage. Specially, within China terrestrial scale a total of 3.456 Pg C was estimated with a depth profile of 0–0.3 m. The variation in MAP and MAT as a result of latitude was primary reason for the spatial differences in SOC content. Under the influence of climatic conditions and soil parent material, SOC content in per-unit volume was the highest in the Qinghai-Tibet plateau region (C6 geographic region), while that of coastal wetlands (C5 geographic region) was the lowest.

## Figures and Tables

**Table 1:** Natural properties of geographical regions.

Geographical Region	Palustrine Wetlands Area × 10^4^ km^2^	Altitude m	MAP mm	MAT °C	Climatic Characteristic
C1	7.5204	327.1	550	1.4	Temperate monsoon climate
C2	0.0329	1847.2	898	18	Subtropical monsoon climate Tropical monsoon climate
C3	0.1595	25.5	1320	16.6	Subtropical monsoon climate
C4	0.0046	43.6	2283	21.3	Subtropical monsoon climate
C5	0.1710	7.2	879	14.1	Temperate monsoon climate Subtropical monsoon climate Tropical monsoon climate
C6	9.9865	3539.1	655	3.3	Plateau mountain climate
C7	0.2493	11.7	510	13.5	Temperate monsoon climate
C8	3.6089	2730	87	4.5	Temperate continental climate

**Table 2:** RDA results between SOC content and environmental variables.

Item	AX 1	AX 2
Eigenvalues	0.579	0.421
Species-environment correlation	0.761	0.000
Cumulative % variance of species	57.90	100.00
Cumulative % variance of species-environment	100.00	0.00
Sum of all canonical eigenvalues	0.579	

**Table 3:** PA results between SOC content and environmental variables

Response Variable	Variable	R^2^	Direct Path Coefficient	Indirect Path Coefficient							Total Path Coefficient	Residual Path Coefficient
				MAP	MAT	Latitude	Longitude	Altitude	Mc	pH		
SOC	MAP	0.999	0.823	--	0.682	−0.618	−0.082	−0.027	−0.003	−0.000	−0.047	0.001
	MAT		0.823	0.682	--	−0.686	−0.118	0.039	−0.011	−0.002	−0.095	
	Latitude		0.856	−0.594	−0.659	--	0.275	0.231	0.009	0.002	−0.736	
	Longitude		0.375	−0.179	−0.259	0.627	--	0.253	0.005	0.002	0.449	
	Altitude		−0.286	0.077	−0.111	−0.692	−0.332	--	−0.013	−0.001	−1.072	
	MC		0.025	−0.088	−0.354	0.322	0.078	0.144	--	0.001	0.104	
	pH		−0.003	0.065	0.427	−0.573	−0.263	−0.140	−0.012	--	−0.496	

Note: ‘--‘ represents that there is no indirect path coefficient between the same environmental factor.
